# Closing the Loop on Polyurethane Foam Waste: Challenges, Emerging Technologies, and the Road to Sustainable Circularity

**DOI:** 10.1021/acssuschemeng.5c12134

**Published:** 2026-02-11

**Authors:** Francisco Velasco, Rocio Villa, Rebeca Salas, Francisco J. Ruiz, Susana Nieto, Jairton Dupont, Eduardo Garcia-Verdugo, Pedro Lozano

**Affiliations:** † Departamento de Bioquimica Y Biologia Molecular B E Inmunologia. Facultad de Quimica, 16751 Universidad de Murcia, Murcia E-30100, Spain; ‡ Departamento de Quimica Organica E Inorganica, 16748 Universidad Jaime I, Castellon E-12071, Spain

**Keywords:** polyurethane Foam, chemical recycling, glycolysis, acidolysis, circular Economy, waste valorization

## Abstract

The increasing production of polyurethane foams (PUFs) and their inherently cross-linked, recalcitrant structure pose major challenges for waste management and circular economy implementation. While mechanical recycling remains the preferred option for thermoplastics, its applicability to thermoset materials such as PUFs is severely limited. Chemical depolymerization has therefore emerged as a key strategy for closing the loop on PUF waste (PUFW). This review provides a critical overview of the chemistry, mechanisms, and technological readiness of the main chemical recycling pathways—particularly glycolysis and acidolysis—highlighting their reaction dynamics, process parameters, and environmental implications. Glycolysis stands out as a mature and versatile technology capable of recovering high-purity polyols under optimized catalytic conditions, whereas acidolysis using (di)­carboxylic acids offers milder operation, faster kinetics, and reduced release of toxic aromatic amines. Hybrid processes that combine both approaches are now entering industrial deployment, as demonstrated by large-scale consortia, such as Renuva, Circufoam, and Recpur, which collectively illustrate the progression from laboratory research to pilot-scale or commercial implementation. Additionally, emerging biotechnological routes—encompassing enzymatic depolymerization and nonisocyanate polyurethane synthesis—and Dynamic Covalent Polymer Networks (DCPNs) approaches are discussed as complementary long-term solutions, though they remain at low technology readiness levels (TRL < 4). Overall, this review identifies the current advances, limitations, and prospects of PUF chemical recycling technologies and provides a roadmap for integrating these strategies into sustainable polymer value chains within a truly circular economy framework.

## Graphical abstract

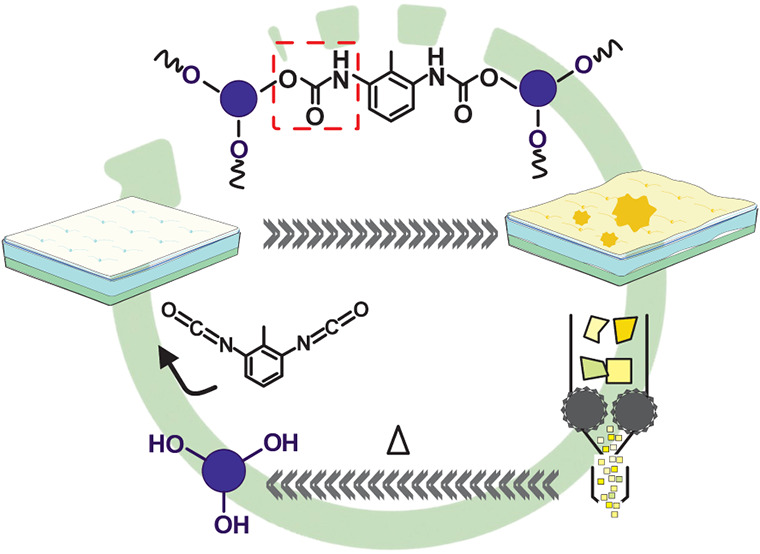

## 1 Introduction

Plastics have revolutionized modern industry and consumers’ lives due to their versatility, durability, lightweight nature, and cost-effectiveness. Since their mass adoption following World War II,^1^ plastics have largely displaced both natural and synthetic alternatives, becoming essential across diverse sectors, such as automotive, construction, packaging, healthcare, electronics, and renewable energy.^2^ Over the past few decades, these materials have maintained a steady “compound annual growth rate” (CAGR) of 3.5–4%. In 2023, plastic production reached 413.8 Mt, of which only 9% proceeded from recycled or biobased materials.^3^ Yet, this global output figure is expected to double by 2050,^4^ with an estimated 121 Mt of mismanaged plastic waste and 3.35 billion tonnes of CO_2_-equivalent emissions projected over the same period.^5^ These alarming trends—widely reported in both academic studies^1,6,7^ and general literature^8–10^ —cannot be solely attributed to polymers or the chemical industry, but also to societal patterns of consumption and disposal that sustain high material throughput. Overall, this underscores the urgent need for technological, regulatory, and behavioral shifts to reverse the current trajectory of plastic consumption and disposal.^11^

Generally, plastic packaging—largely dominated by polyolefins and polyesters—rules global waste streams due to short product life cycles.^12^ This area of expertise encompasses mainly thermoplastics, whose physicochemical properties enable easy melting and reshaping, favoring their mechanical recycling.^13^ The following recycling outlook analysis of such End-of-Life (EoL) products clearly exemplifies the current worrying and out-of-step situation of plastic waste management models.

In 2018, the Ellen MacArthur Foundation and the UN Environmental Programme launched the *Global Commitment*, a voluntary effort to tackle plastic waste and pollution.^14^ This agreement gathered over 55 government signatories (i.e., France, Australia, United Kingdom, *etc*.) and 1,000 assorted organizations (i.e., Indorama Ventures, Coca-Cola Company, Veolia, *etc*.). Altogether, these signatories represent approximately 20% of global plastic packaging production, usage, and/or supply.^14^ Table 1 presents a summary of their progress since the initiative’s launch.^15^ Despite the ambition behind the *Global Commitment*—proven by the removal of plastics, such as polyvinyl chloride (PVC), expanded polystyrene (EPS), or extruded polystyrene (XPS), by these companies (see column 6)—most targets still remain underdeveloped. The reduction of virgin plastic consumption and the integration of postconsumer recycled (PCR) content in new products are significantly below the target (see columns 2 and 3, respectively). Although more concerning is the decline in the use of reusable packaging, which has dropped by more than half (column 4).

**Table 1:** Progress on the Global Commitment Targets (2019–2024)^15^

Year	^a^VPR (%)	^b^PCRc (%)	^c^RP (%)	^d^R/C Packaging (%)	^e^PPR
**2019**	0.0	4.7	2.9	60.0	60.0
**2020**	0.1	6.2	1.9	65.0	69.0
**2021**	2.1	8.2	1.8	65.4	80.0
**2022**	0.1	10.0	1.2	64.5	92.0
**2023**	0.1	11.7	1.2	64.5	>99.9
**2024**	4.0	14.0	1.3	70.0	>99.9
**2025 target**	**21.0**	**26.0**	-	**100.0**	**100.0**

a VPR: Virgin Plastic Reduction.

b Postconsumer Recycled Content.

c Reusable Packaging.

d Recyclable/Compostable Packaging.

e Problematic Plastics Removal (PVC, EPS, XPS).

Despite the unsatisfactory results presented in the 2024 progress report by the Ellen MacArthur Foundation, the broader outlook is likely more overwhelming. This case study of the *Global Commitment* signatories encompasses mainly organizations and governments with heightened environmental awareness and more stringent regulatory frameworks and infrastructure. Thus, the performance of the remaining global plastic value chain—especially in regions with weak enforcement or limited resources—may be significantly worse across all key indicators. In this sense, a concerning reflection of this broader outlook is the lack of a binding agreement within the Intergovernmental Negotiating Committee (INC) on Plastic Pollution, mandated by UNEA resolution 5/14 to deliver a legally binding treaty that addresses the full life cycle of plastics—from production and design to use and end-of-life.^16^ Despite multiple negotiating rounds since 2022, the INC has yet to deliver a consensus. The impasse is largely attributed to deep divisions over the treaty’s scope, whether it should cap virgin plastic production or focus instead on downstream measures such as waste management and product design.

The limited success of this voluntary framework illustrates the gap between the policy ambition and systemic capability. As a visual example, Figure 1 depicts a Sankey diagram that shows the worrying life cycle of plastic packaging.^17^

**Figure 1:**
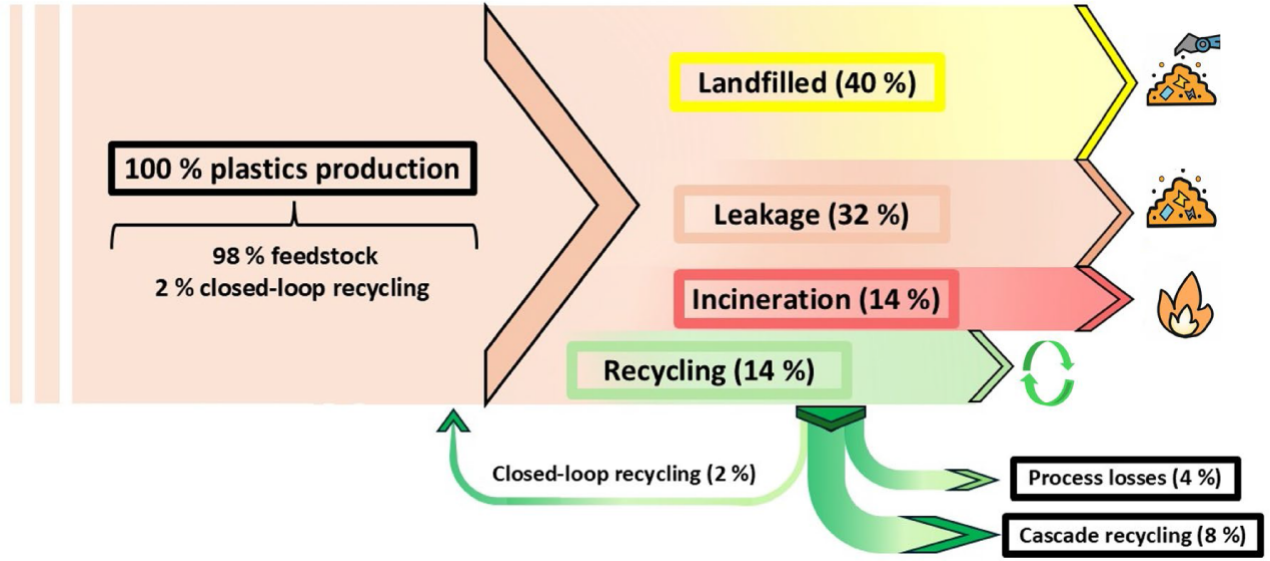
Sankey diagram of the production and waste management of plastic packaging.^14^

Also, it is worth noting that if such a gap persists for packaging, an even more pronounced and critical situation arises for polymers used predominantly in long-lived heterogeneous applications. In this context, polyurethanes (PUs) are a paradigmatic example, as they represent a distinct challenge due to their thermoset nature, long service life, and additive-rich formulations.^18^ Despite being a profitable market, which exceeded USD 75 billion in 2022 and is expected to reach over USD 100 billion by 2030, its productive recycling is nonexistent.^19^ Flexible and rigid foams represent a major share of PU demand, providing lightweight thermal insulation and comfort materials in buildings, appliances, furniture, and automotive seating.^20^ However, such bulky materials generate a complex waste stream, which lacks efficient, sustainable, and profitable waste management strategies, thus historically being disposed of via landfilling, incineration, or conversion into low-value end-products.^21,22^

Structural barriers, such as the cost disparity between virgin and recycled plastics, inadequate waste segregation systems, and the complexity of multilayer materials, continue to hinder progress. Without mandatory regulations, financial incentives, and significant investment in infrastructure and innovation, such voluntary efforts are unlikely to reach their targets.^23–26^

This review deliberately narrows its scope to the recently mentioned PU case to provide a more focused and critical analysis, as this polymer ranks first in thermoset plastics’ production and sixth in global plastics manufacture.^3^ While numerous studies address the broader spectrum of plastic recycling,^27–30^ this review focuses first on the difficulties that waste management technologies face with thermoset polymers, and how these constraints are shifting toward chemical recycling, and more specifically, toward selective depolymerization. Thus, the present work seeks to provide a critical and integrative perspective on PUF waste management by identifying the current advances, limitations, and prospects of this material’s chemical recycling. The following section perfectly illustrates how widely used techniques in thermoplastics’ recycling are profitless in contaminated waste streams or even useless in more recalcitrant and cross-linked polymers (e.g., PUF).

## 2 Mechanical Recycling Limitations and the Urgent Need for Circularity

The previous subset analysis of plastic packaging pertains to thermoplastics, such as polypropylene, high-, low-, and linear low-density polyethylene, and polyethylene terephthalate (PP, HDPE, LDPE, LLDPE, and PET, respectively), which collectively represent the bulk of consumer plastic use and disposal.^31^ Despite the low recycling rates discussed above, these polymers’ recyclability is significantly higher than that of thermosets,^32^ which are more mechanically, chemically, and thermally stable.^33,34^ The molecular network of these polymers prevents them from being processed by melting or dissolution processes, hampering mechanical recycling.^13,31^

This recyclability is strongly influenced by the polymer chain linearity and cross-linking degree, which eventually define its degradability and processability.^35^ In this sense, mechanical recycling is a cost-effective and efficient solution for managing many postproduction wastes and EoL thermoplastics, motivating such technologies to be developed and integrated into industrial processes.^36^ Additionally, both Life-Cycle Assessment (LCA) and Techno-Economic Analysis (TEA) tools have evinced that mechanical recycling can be a sustainable and profitable approach when applied under appropriate conditions.^37–40^ For instance, Cosate de Andrade et al.^40^ determined the LCA analysis of poly­(lactic acid) (PLA) waste management, concluding that the mechanical recycling of this polymer presented less environmental impact in comparison to chemical recycling or composting. Similarly, Schyns and Shaver^39^ reviewed the relevance of mechanical recycling in plastic packaging and the major advancements over the last 50 years, confirming its need in polymer waste management.

Yet, mechanical recycling presents its own set of trade-offs. In this sense, most EoL products consist of mixed streams (i.e., multipolymers, inorganic contaminants, organic wastes, *etc*.)^33,41^ that significantly influence processing efficiency.^37,42^ This inherent heterogeneity limits the purity and performance of recycled outputs, particularly in sectors with strict regulatory requirements such as food packaging and medical devices, as highlighted by some organizations, such as the Food and Drug Administration (FDA).^31^ To meet these standards, mechanical recycling systems should incorporate more complex and costly pre- and/or post-treatments, which end up limiting their realistic application.^13,31,41^

In thermosets—the other major class of plastics—the handicap is even more evident. Mechanical recycling of these polymers entails downcycling, leading to an irreversible degradation of the final material properties and a profitability drop.^34,37,43^ Thus, thermosets can only undergo mechanical recycling a reduced number of times, eventually losing their function and being discarded.^22^ To avoid this downcycling, most authors agree the best solution is the implementation of “more complex technologies” to recover the raw materials and close the loop.^44–46^

These “more complex technologies” refer primarily to tertiary or chemical recycling processes, which encompass various depolymerization and conversion routes designed to recover the chemical value of plastics.^43^ Such methods enable the cleavage or exchange of specific chemical bonds, allowing the complete recovery of petrochemical feedstocks (as in pyrolysis or gasification) or the regeneration of original monomers (as in glycolysis or hydrolysis).^47^ Unlike mechanical recycling, these processes are aimed at avoiding structural degradation and malfunction associated with downcycling, thereby promoting a truly circular model of production and consumption, which implies a noteworthy reduction in fossil-based feedstock extraction.^48^

This shift toward chemical recycling is not only a technological necessity but also an environmental imperative. If current trends continue, plastic production will account for up to 20% of global petroleum consumption by 2050.^7^ In response, significant investments are being directed toward chemical recycling infrastructure, particularly in Europe, where the market is projected to attract €8 billion by 2030, which means a 300% increase over 5 years.^49^ The integration of chemical recycling approaches into global waste management systems represents both a sustainability milestone and a substantial economic opportunity for the polymer industry.^11^

## 3 Polyurethane Foam Market Trends

As mentioned above, chemical recycling approaches are particularly important for thermoset polymers due to their inherently recalcitrant nature. These plastics account for 15–20% of global plastic production^3^ and include epoxides, unsaturated polyesters, rubber materials, and most PUs.^35^ PU was first synthesized by Otto Bayer in 1937 ^50^ and is of special interest, as it ranks sixth in plastic production worldwide.^3^

Owing to their unique properties, PUs are used in a broad range of applications, including coatings, adhesives, insulation panels, foams, *etc*.^51^ The PU market is projected to grow at a CAGR of ∼5% between 2021 and 2028,^52^ with an overall production rise of 25% by 2030.^53^ This manufacturing and investment increase is primarily driven by the development of energy-efficient buildings in the construction sector.^54^

In parallel with the steady rise in PU production and consumption, scientific interest in the EoL management of this material has grown markedly in recent decades. This trend is clearly reflected in the number of peer-reviewed publications addressing “recycling AND polyurethanes,” as indexed in the Web of Science database between 2001 and 2025 (see Figure 2).

**Figure 2:**
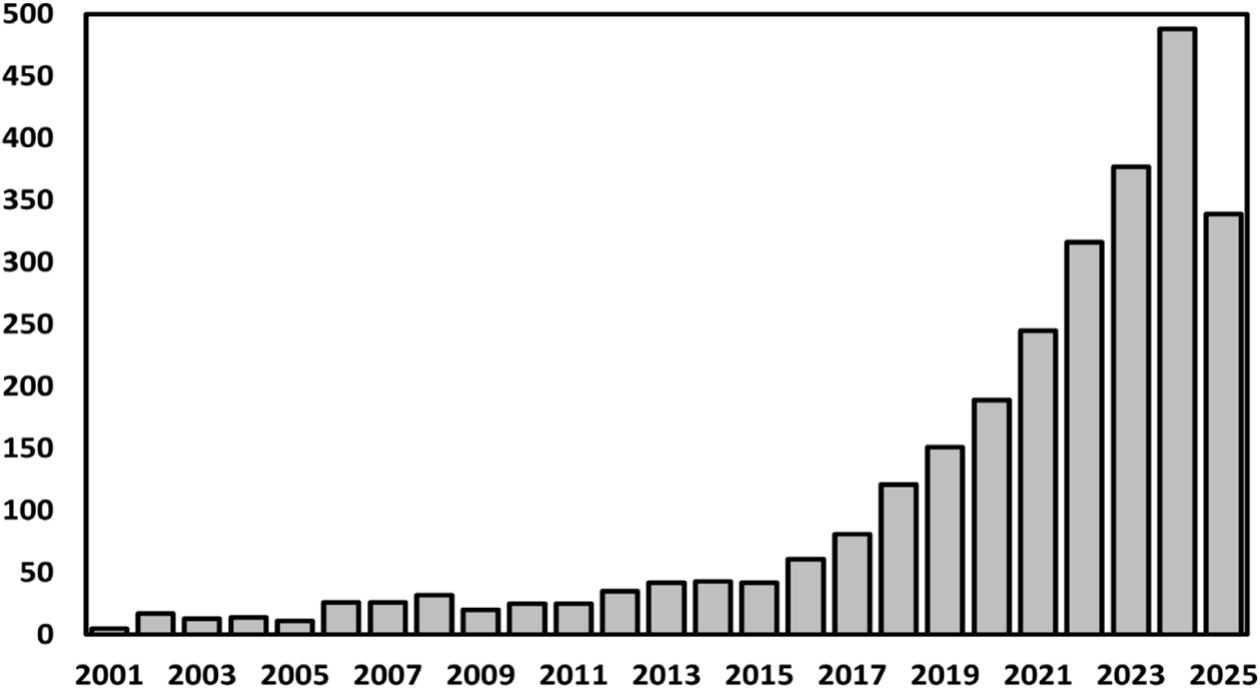
Peer-reviewed publications addressing “recycling AND polyurethanes,” as indexed in the Web of Science database between 2001 and 2025.

## 4 Polyurethane Foam Chemistry

The growing market demand for PU is closely associated with the widespread use of PUFs, which account for over two-thirds of the total PU production.^51^ This dominance is largely driven by the versatility imparted by their remarkable chemistry, enabling their use across sectors such as comfort applications (i.e., mattresses, furniture), the automotive industry (e.g., seating), and construction (e.g., insulation panels).

Unlike thermoplastic PU, PUFs show a complex molecular architecture and high crystallinity.^55^ The preparation of this material consists of polyaddition reactions between isocyanates and polyols, which confer its distinctive versatility and resilience.^56^ Both the selection and the ratio of these monomers—alongside catalysts, blowing agents, low-molecular-weight chain extenders, or additives—determine the final properties of the resulting material.^57–59^ Table 2 collects commonly used reactants in PUF synthesis and their respective roles and impact on the final properties of the product.^60^ For instance, polyol functionalization and cross-linkers mainly define the application of the final material (e.g., rigid or flexible PUF), owing to density and thermal properties (entries 2 and 6). Moreover, the blowing agent and surfactant ratio are key elements to avoid collapse and control cell formation (entries 3 and 4). Finally, the use of additives (i.e., fillers, antioxidants, *etc*.) provides specific properties to the final material, thereby determining its practical utility and commercial value (entry 7).

**Table 2:** Common Reagents Used in the Preparation of PUFs and Their Respective Influence on the Properties of the Final Material^a^

Entry	Reactant	Function
**1**	**Isocyanate**	Determines PUF reactivity and provides rigidity and strength to the final material. Generation of secondary bonds responsible (i.e., urea, biuret, allophanate, isocyanurate, *etc*.).
**2**	**Polyol**	Main reactant along with isocyanates. Determines flexibility, density, and thermal properties of the PUF.
**3**	**Blowing agent**	Provides the foam structure. It affects the cell size and structure, thus impacting in the foam density.
**4**	**Surfactant**	Key component for stabilizing the foam cells during expansion. It controls cell size and structure while preventing collapse during synthesis.
**5**	**Catalyst**	Boosts the reaction between isocyanates and other substrates (i.e., polyol, blowing agent, *etc*.).
**6**	**Chain extender/cross-linker**	Enhances mechanical strength and hardness.
**7**	**Additives**	Affect specific properties, such as flame retardancy, color, UV resistance, thermal conductivity, or stiffness.

a Information provided by public–private partners (*e.g.,* Technological Centre for Wood and Furniture of the Region of Murcia (CETEM)^62^ and INTERPLASP S.L.).^63^

As can be seen in Figure 3, the presence of water, along with the stoichiometric excess and high reactivity of diisocyanates, can lead to aromatic, amine, biuret, allophanate, or even isocyanurate linkages. These groups are present in the PUF backbone along with urethane bonds, providing a heterogeneous-recalcitrant network that is hardly (bio)­degradable.^55^ The complexity of PUF’s molecular architecture presents an important barrier to recycling. In particular, the cleavage of carbamate bonds is hindered by steric hindrance, chain rigidity, and hydrophobic interactions, thereby limiting the efficiency of depolymerization pathways.^61^

**Figure 3:**
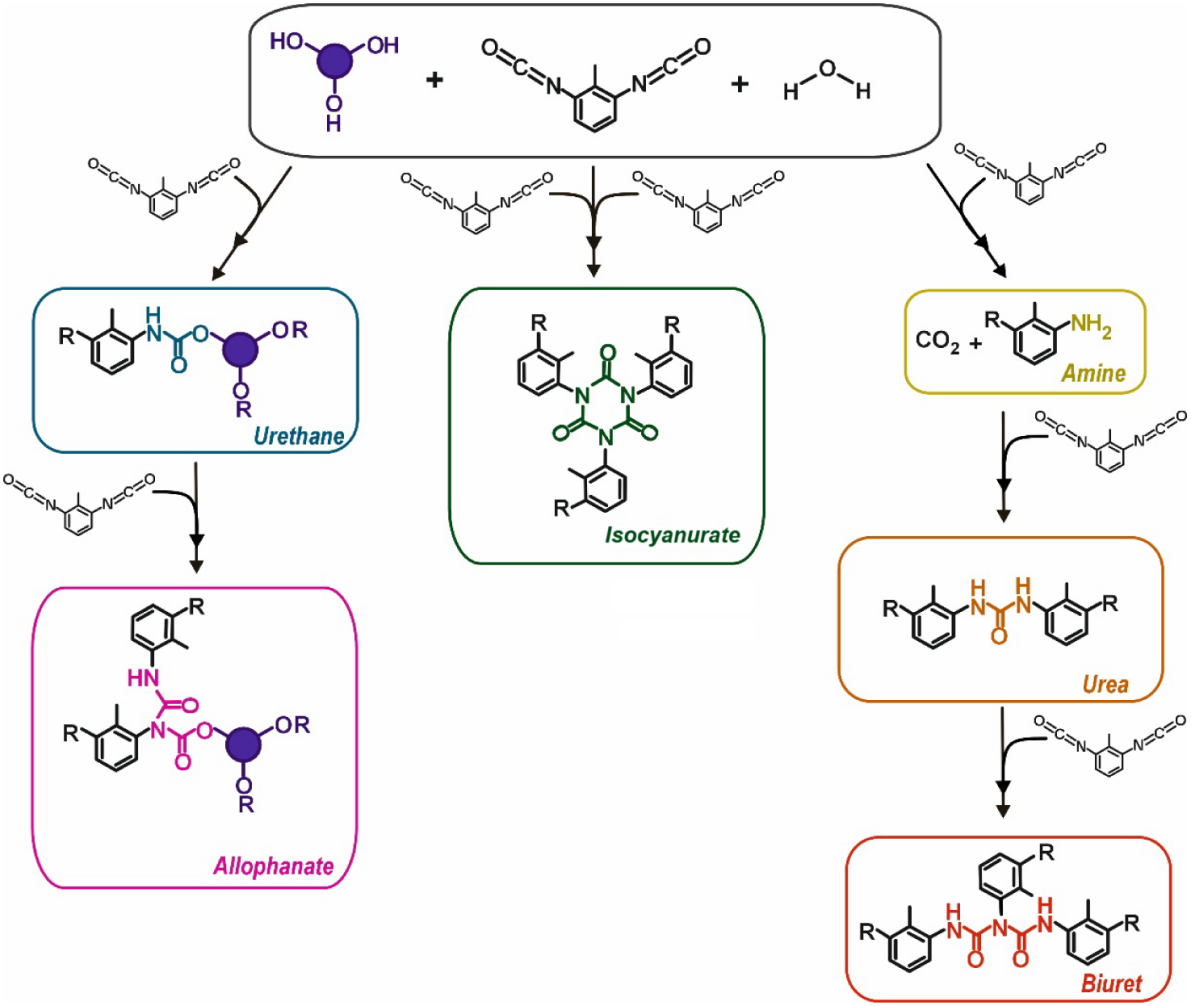
A schematic description of the different functional groups formed during PUF synthesis.

The remarkable expansion of the PUF market, combined with its thermostable nature, underscores the problem regarding the lack of effective EoL management strategies and causes a serious environmental and economic challenge. These concerns are shared by researchers, governments, and industry stakeholders alike, highlighting the urgent need to develop suitable and efficient recycling technologies capable of reintroducing PUF waste (PUFW) into the material value chain.^64^

## 5 Polyurethane Foam Waste Environmental Concerns

Nowadays, most PUFWs are disposed of via landfilling or incineration.^22^ According to the economic research institute CSIL (Center for Industrial Studies), the European production of mattresses reaches around 50 million units annually, accounting for 50% of the PUF production.^65^ Additionally, it is estimated that every year more than 40 million mattresses are discarded only in the EU—equivalent in height to 904 stacked Mount Everests.^21^ To combat this environmental damage, several EU countries (i.e., the Netherlands, Denmark, Sweden, Austria, *etc*.) have adopted strict restrictions on the disposal of urban waste and landfilling.^66,67^ However, the absence of efficient PUFW management strategies makes energy recovery (e.g., incineration) the main solution in these countries.

Furthermore, this problem goes beyond EoL products, since postproduction waste also represents a significant challenge for PUF manufacturers (see stage 2, Figure 4). Typically, 10–15 wt % of the final product is discarded as scraps and cutoffs, substantially decreasing overall process efficiency and sustainability.^68^

**Figure 4:**
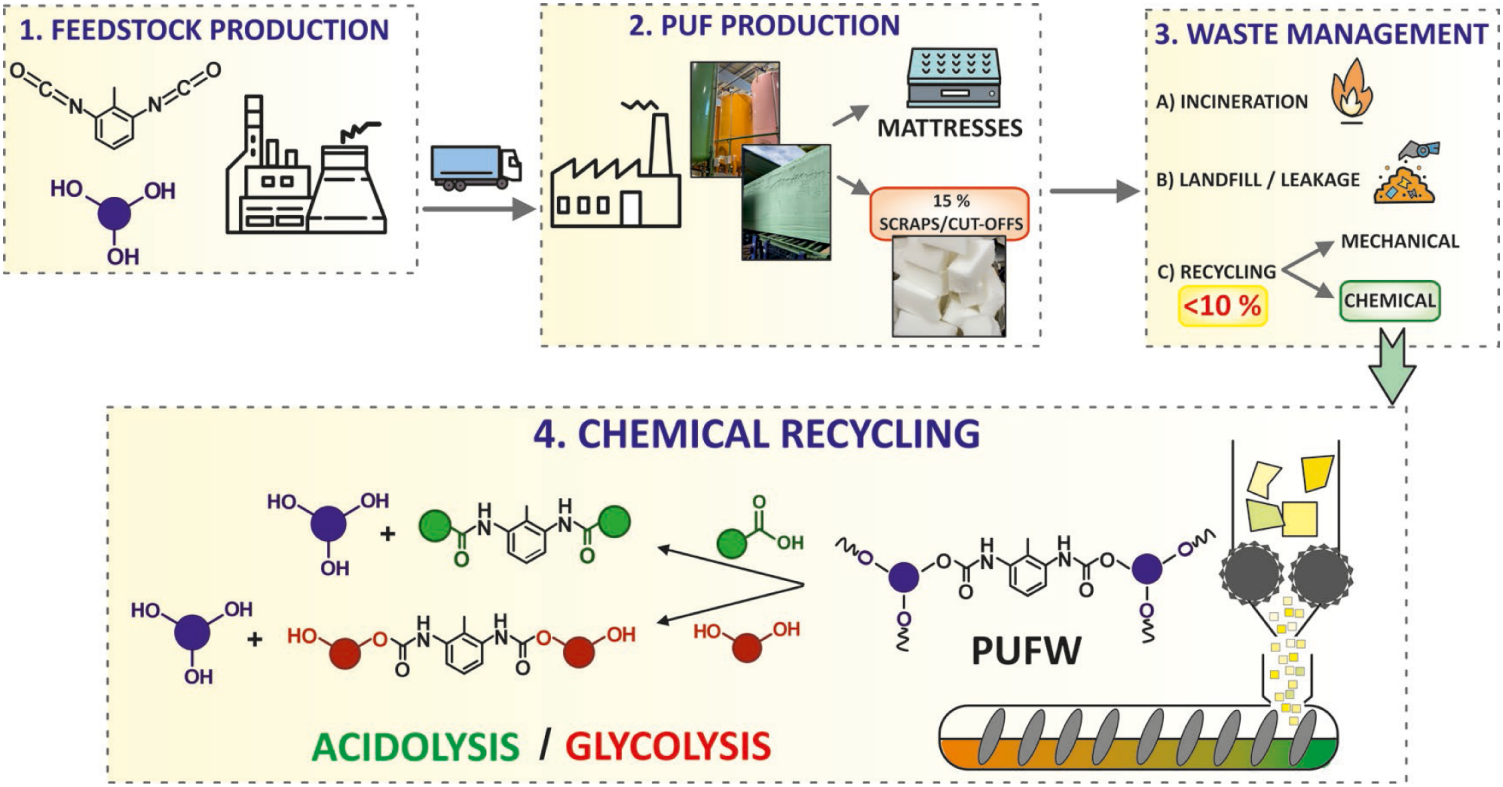
Production and waste management chain values of PUF. **1.** Manufacture of the main raw materials employed during PUF synthesis (e.g., (di)­isocyanates and polyol). **2.** PUF slabstock line and final products (e.g., postproduction waste and foam for mattresses). **3.** Main routes for postproduction and postconsumer PUFW. **4.** Ideal PUFW management via chemical recycling.

This inherent material loss arises from foam expansion against the reactor boundaries, which leads to the formation of a dense, rigid outer layer—commonly referred to as “peel”—that lacks commercial value. This economic loss, alongside more stringent policies, motivates the development of the previously mentioned chemical recycling technologies. In this sense, Figure 4 depicts the overall production and waste management chain value of PUF, wherein the manufacturing is displayed in stages 1 and 2, while stage 3 exhibits the current waste management state regarding postproduction and EoL PUFW. Lastly, stage 4 illustrates the chemical recycling of PUFW through a two-step methodology, in which a grinder is connected to a plug-flow reactor.

However, it is important to note that EoL foams face an important barrier to recycling, which postproduction PUFW does not: contaminants. Postconsumer waste streams are heterogeneous multilayer materials that are frequently contaminated and often include textile compounds, adhesives, and dust originating from mattresses, furniture, or insulation assemblies. Also, they contain inorganic and organic residues that are not inert and play a decisive role in determining process robustness at scale. From an industrial perspective, inorganic fillers and certain additives can promote catalyst deactivation or poisoning, particularly in acid- or metal-catalyzed depolymerization routes. Similarly, organic residues and additives, as well as degradation and oxidized products, can affect color, odor, viscosity drift, and overall stability of the recovered polyols, oligomers, or amines, complicating formulation control and limiting direct reintegration into high-performance foam applications. Residual solids and extractives may further impair cell morphology, mechanical integrity, and long-term aging behavior of regenerated foams, thereby constraining product qualification and market acceptance. Such issues are largely absent in postproduction PUFW, which explains why laboratory-scale studies often report higher selectivity and reproducibility than those observed when processing real postconsumer streams. Additive leaching, migration, and chemical transformation during service life further exacerbate this discrepancy, as documented for polyurethane materials and plastic additives under environmental exposure.^28^ Consequently, industrially viable PUFW recycling technologies must be inherently tolerant to contaminants or integrate effective pretreatment and separation strategies to ensure consistent polyol quality and stable process operation.

## 6 Polyurethane Foam Chemical Recycling Technologies

As mentioned above, tertiary (chemical) recycling stands out as a feasible alternative to surpass the limitations of mechanical recycling.^69^ This prominent technology is classified depending on the final product obtained.^70^ Thermal energy-driven depolymerization encompasses pyrolysis, gasification, or hydrogenation and leads to the recovery of refined monomeric compounds (i.e., hydrocarbons, fuel, syngas, *etc*.),^71,72^ decoupling polymer prices from oil-derived raw material costs.

However, the sizable energy inputs and low overall economic profits encourage the design of milder approaches, which enable the straightforward conversion of EoL products into the initial monomers, that is, the design of selective depolymerization routes.^46,73^

The depolymerization approaches rely on nucleophilic agents—such as water, alcohols, and amines—used either in the presence or absence of catalysts. Depending on the nature of the cleavage agent involved, the reactions are commonly classified as hydrolysis (water), alcoholysis (alcohols, e.g., methanolysis or glycolysis), aminolysis (amines), acidolysis (acids), or hydrogenolysis (hydrogen).^74^ Each pathway promotes bond scission through distinct nucleophilic or reductive mechanisms, ultimately determining the type, purity, and functionalization of the recovered products (Figure 5). Typical catalysts include organic metal salts (e.g., Zn­(OAc)_2_), alkali metal hydroxides (e.g., NaOH), heterocyclic amines *(e.g.,* 1,4-diazabicyclo[2.2.2]­octane (DABCO)), or metal carboxylate salts (e.g., tin­(II) 2-ethylhexanoate). In the context of PUFs, the main commercial and research interest has been directed toward the efficient recovery of polyols suitable for repolymerization. In contrast, only a limited number of studies, mainly at the academic level, have focused on the recovery of diisocyanates or their corresponding aromatic diamine precursors.^75–77^ In this sense, Figure 6 depicts the breakage routes and chemical mechanisms of the most common depolymerization approaches reported.

**Figure 5:**
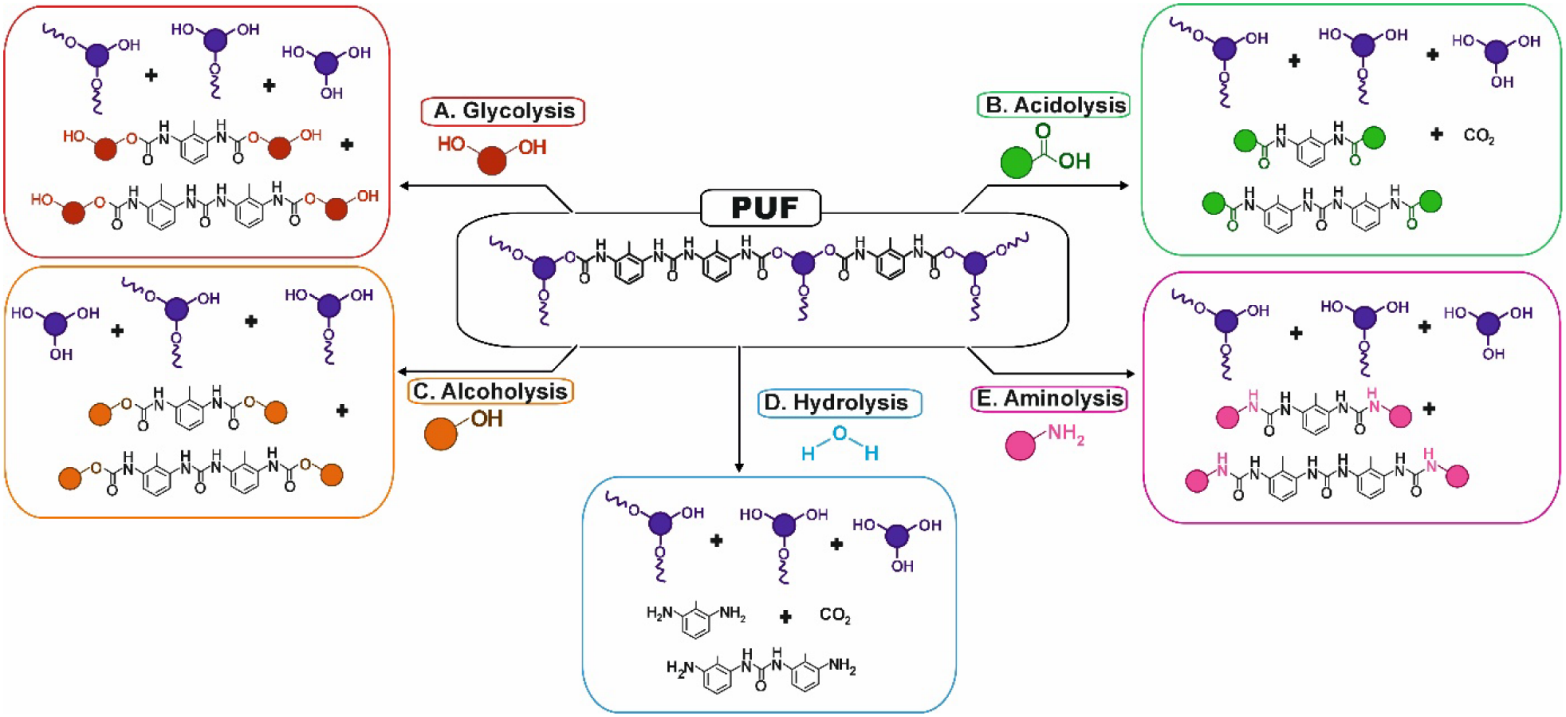
Main recycled products obtained from different depolymerization approaches of PUFW, namely: A) glycolysis, B) acidolysis, C) alcoholysis, D) hydrolysis, and E) aminolysis.

**Figure 6:**
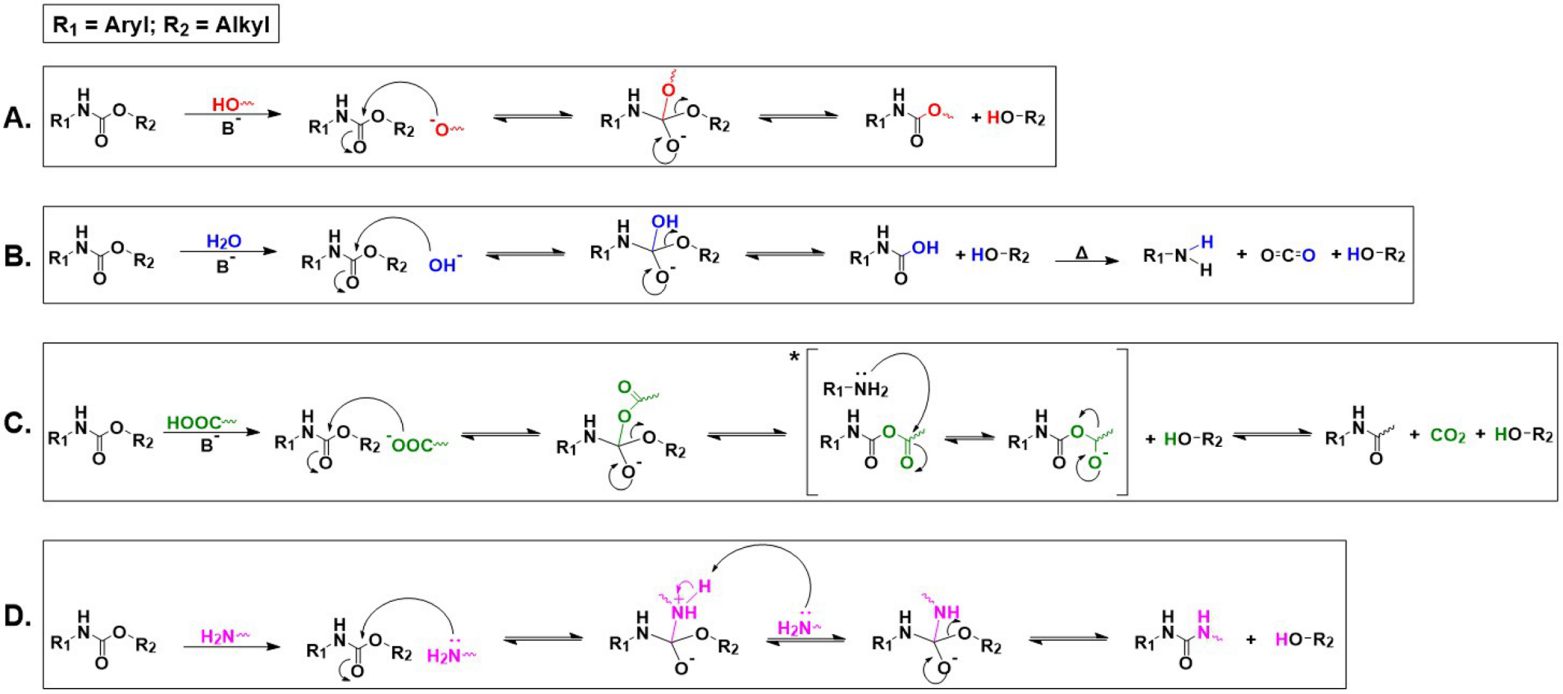
Reaction mechanism proposal of different depolymerization approaches, namely: A) alcoholysis, B) hydrolysis, C) acidolysis, and D) aminolysis. *C: The proposed mechanism, previously reported by Bech et al.,^77^ consists of amide formation during polyurethane acidolysis via a mixed carbamic–carboxylic anhydride intermediate. Intramolecular nucleophilic attack of the nitrogen lone pair on the activated carboxylic acyl carbon generates a tetrahedral intermediate, followed by collapse with expulsion of the carbamate-derived leaving group. Subsequent proton transfer yields a thermodynamically stable amide and regenerates the carboxylic acid functionality. This pathway explains the effective “fixation” of amine fragments as solid amide byproducts during acidolysis.

The studies discussed below are representative examples illustrating the latest advances of each corresponding depolymerization strategy. Thus, the purpose is to show broader mechanistic and technological trends, as well as frequent constraints, rather than an exhaustive survey, as such comprehensive overviews have recently been provided.^74,78^ Hydrolysis (Figure 5D) is among the earliest strategies applied to PUFW—initially tested in the USA automotive industry during the 1970s.^79^ Nevertheless, it was quickly deserted due to the harsh operating conditions (e.g., temperatures of 190 to 400 °C and up to 40 bar of pressure) and the undesired byproducts released during the reaction (e.g., free-aromatic (di)­amines).^80–82^ Recent studies have demonstrated that PUFW can be efficiently depolymerized via hydrolysis using superbase catalysts in combination with ionic liquids.^83,84^ This approach enables effective depolymerization at temperatures below 100 °C, which are significantly milder than those previously reported in the literature, with an impact on the quality of recovered monomers. As a result, the recycled polyol could be incorporated up to 40% into the virgin polyol to synthesize new PUF. Additionally, the company Evonik Industries has recently published some patents that combine efficient pretreatments along with hydrolysis mechanisms to recover virgin-grade polyols.^85,86^

Similarly, alcoholysis (Figure 5C) depolymerization processes involve the substitution of one hydrogen atom of water by the aliphatic chain of an alcohol.^72^ Despite several patented approaches, industrial viability remains limited due to high energy demands and challenges in product yield and purity.^59,82^ To solve this problem, mechanocatalytic methanolysis under mild reaction conditions (below 100 °C) has been recently explored.^87^ A novel strategy combines NaOH-impregnated PUF and a Cu/MgAlO_*x*_ cocatalyst (20 wt %) along with mechanochemistry to reach polyol yields over 80% in 90 min. This example highlights the potential of combining urethane exchange reactions with mechanochemistry and catalyst technology.^88–90^ Yet, although these processes are opening new pathways for unexplored—even seemingly “impossible”—chemical reactions,^91^ several drawbacks regarding scalability, continuous processing, sample contamination (e.g., ball mills), or kinetics control (e.g., temperature constraints) may hinder their industrial implementation.^92^

Alternatively, aminolysis (Figure 5E) is a relatively novel chemical depolymerization strategy that relies on the nucleophilic attack of amines on carbamate bonds, leading to the formation of urea groups as the exchange between the urethane and the amine groups proceeds.^51^ In a notable and recent study, Grdadolnik et al. proposed a below-1-h two-step microwave-assisted aminolysis at 220 °C employing tris­(2-aminoethyl)­amine (TREN), capable of degrading >99% of the urethane groups present in the PUFW.^93^ TREN acts as both a nucleophile and a catalyst, showcasing the multifunctional role that amines can play in these depolymerization systems. In another recent study conducted by Olazabal et al.,^82^ different commercial PUFs were depolymerized by combining 2-(methylamino)-ethan-1-ol as a nucleophile and an acid:base mixture as a catalyst, based on triazabicyclodecene and methanesulfonic acid (TBD:MSA). The results demonstrated complete depolymerization with high yields of diurea oligomers at 160 °C and reaction times as long as 3 h. However, cross-linked rigid PUF did not undergo depolymerization. In another research, Van Belleghem et al. managed to effectively carry out the ammonolysis of cross-linked rigid PUF at different reaction conditions, obtaining a volumetric productivity of up to 300 g·L^–1^·h^–1^ at 180 °C in a 10:1 NH_3_ molar excess relative to the number of isocyanate-derived groups in the rigid PU sample.^94^

Despite the recent and promising results of this depolymerization strategy, these processes present technical and economic challenges: (i) the high amine reactivity (commonly used in PUF synthesis) can lead to cross-linking side reactions and rearrangements along the polymeric chain (e.g., repolymerization),^74,82^ (ii) additional costs derived from the excess of amine/ammonia cleavage reagents substantially reduce the economic viability of the overall process,^87,95^ and (iii) disubstituted urea byproducts increase recycled product viscosity and stiffness,^74,96^ limiting the final scope of the recycled polyol. Consequently, aminolysis approaches remain at low TRL and are industrially nonviable.

Among emerging strategies, acidolysis (Figure 5B) has recently gained attention for its selective cleavage of urethane bonds.^97^ Although initially explored in the 1990s using inorganic acids, such as HCl,^74,98^ renewed interest has been driven using (di)­carboxylic acids (DAs), such as adipic or succinic acid as nucleophilic agents.^97^ The main advantages lie in the selective urethane cleavage, yielding recycled polyols devoid of toxic byproducts (e.g., free-aromatic (di)­amines).^99–101^ The low content of aromatic amines generated by the urea cleavage is “fixed” by the acidolysis agent, which reacts with those byproducts to form amides.^78^ However, the presence of solid amides deteriorates the recycled polyol quality,^78^ triggering the conduction of multistage protocols.^95^ Moreover, its operational flexibility allows it to work with or without solvents (e.g., vapor-phase acidolysis) and catalysts (e.g., Lewis acids such as Zn­(OAc)_2_ or ZnO) at mild-to-moderate temperatures (as low as 140 °C).^102,103^ However, its relative novelty implies that several parameters remain under research, such as the influence of the acid structure or ratio with respect to PUF.^104^ Additionally, other concerns encompass side reactions (e.g., esterification of polyol hydroxyl end-groups) and product variability (highly dependent on PUF composition).^37,98^

Lastly, among all depolymerization strategies, glycolysis (Figure 5A) remains the most extensively studied and technologically mature.^22,73,75,98,105^ While glycolysis is, in essence, a subset of the broader alcoholysis category, it is typically treated as a different process within the field. This distinction arises from its exclusive use of diols/glycols as nucleophilic agents, which confer unique reactivity profiles and product distributions due to their properties (i.e., viscosity, vapor pressure). The use of lower-energy alcohols, such as methanol, lacks industrial attractiveness, as these depolymerization routes require pressurized operation, complex separations, and enhanced safety measurements. In contrast, glycolysis is favored at scale due to its direct compatibility with polyol reuse and integration within existing polyurethane production frameworks.

The glycolysis mechanism consists of a transcarbamoylation reaction, which can occur via two mechanisms—associative or dissociative—leading to carbamate oligomers and original polyols (Figure 7).^90,106^ In the presence of free hydroxyl groups, associative transcarbamoylation proceeds via a classical nucleophilic displacement pathway (Figure 7A). In contrast, dissociative transcarbamoylation under analogous conditions may proceed through an elimination-addition sequence, in which the urethane first undergoes retroformation to the corresponding isocyanate and alcohol, followed by subsequent exchange with a second alcohol (Figure 7B).

**Figure 7:**
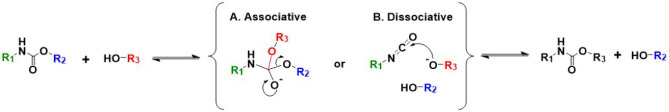
Transcarbamoylation of the urethane group via both associative (A) or dissociative (B) mechanisms proposed by Bakkali-Hassani and coworkers.^106^

This methodology offers significant versatility, being applicable to both rigid and flexible foams.^107–111^ Additionally, the chemical flexibility of this technology is shown by the wide range of catalysts and nucleophiles available.^98,107,112^ Regarding the process, two glycolysis methodologies can be performed depending on the glycol-to-PUFW mass ratio and the type of glycol employed, namely split-phase and one-phase glycolysis.^98^ While split-phase glycolysis procedures lead to higher-quality recovered polyols,^113^ the glycol molar excess required to obtain two distinct phases greatly hinders the overall process profitability, limiting its industrial implementation to date.^105,114^

Overall, glycolysis’s main drawbacks are the obtention of a complex mixture of oligomers (e.g., flexible and rigid segments) and the high energy consumption due to temperature conditions (e.g., 170–250 °C). These harsh conditions can promote undesired side reactions, namely (i) cracking of allophanate and biuret groups, (ii) transesterification of the urea group, (iii) C–N linkage cleavage of the carbamate bond instead of C–O scission, and (iv) thermal degradation of main bonds.^78,113,115–117^ However, it has recently been reported that a sustainable and scalable glycolysis approach with negligible toluene diamine (TDA) content (<0.05 wt %).^118^ In this work, typical glycolysis constraints are avoided due to the optimal combination of two ionic liquids (ILs),1-*n*-butyl-3-methylimidazolium chloride ([Bmim]­[Cl]) and 1-*n*-butyl-3-methylimidazolium acetate ([Bmim]­[OAc]), which enable efficient depolymerization under soft reaction conditions (<100 °C, 1 atm, 4 h). Additionally, this approach has been successfully tested (depolymerization yield >95%) with flexible and rigid PUFs, as well as a mixed waste stream composed of different PUF postconsumer products, proving the robustness, versatility, and technical feasibility of this technology at the laboratory scale. Although this IL-assisted glycolysis enables high selectivity under mild conditions, important limitations must be acknowledged. Imidazolium-based ionic liquids, particularly early-generation salts, such as [Bmim]­[BF_4_], are not intrinsically sustainable, and life-cycle assessments have shown that several members of this class can exhibit substantially higher environmental impacts than conventional molecular solvents when evaluated on a mass basis using current production routes.^119,120^ Accordingly, ILs should be regarded as performance-driven functional materials rather than universal green solvent substitutes, with their deployment justified only where clear system-level benefits outweigh their documented environmental burdens. In this sense, the process design and recovery of such media are essential to mitigate both cost and environmental impact and to align more closely with Circular Economy principles. Overall, the use of ILs should presently be viewed as proof-of-concept media rather than direct drop-in industrial solvents.

## 7 Academic and Industrial Pathways for Polyurethane Foam Chemical Recycling: Glycolysis vs Acidolysis

As previously outlined, the chemical depolymerization of PUFW has emerged as a critical strategy for closing the loop on PUF-based products. Among the array of available technologies, glycolysis and acidolysis have gained prominence as the most promising pathways due to their relatively high selectivity, operational flexibility, and valorization potential.^73,74,78,98^ Each approach offers distinct mechanistic pathways and technological readiness, but their comparative efficacy remains a topic of ongoing investigation across academia and industry.

Since the introduction of dicarboxylic acids (DAs) as cleavage agents, acidolysis has achieved what, until recently, seemed unlikely: challenging the long-standing dominance of glycolysis in the academic landscape of PUF depolymerization. This emerging competitiveness has opened a growing debate over the relative merits of each approach, particularly in terms of selectivity, product quality, and compatibility with downstream valorization routes. As will be discussed in this section, this academic interest has translated into tangible industrial momentum, with several key consortia and commercial initiatives adopting acidolysis as either an alternative or a complementary strategy to glycolysis (see Table 3).

**Table 3:** Industrial Consortia to Close the Loop of Post-Consumer Mattresses and Insulation Panels (2020 Onwards)

**Consortium (1)**	**Renuva^TM^**
**Process**	The starting foam is compressed, degassed, and combined/soaked with a polyol in a nonbackmixing tubular reactor (e.g., plug-flow reactor). Subsequently, the digestion is carried out *via* glycolysis-acidolysis approach.^142^
**Partnership**	Dow Chemical Company, Ecomaisson, Orrion Chemical Orgaform, H&S Anlagentechnik, and Vita Group.^143,144^
**TRL**	Industrial implementation and semi-industrial scale applications (8), with a 200,000 mattresses processing capacity per year.^143,144^
**Plant location**	Semoy, France.^143,144^
**Patents**	WO 2025/075820 A2 (Dow)^142^ & WO 2018/091568 A1 (H&S).^145^
	
**Consortium (2)**	**Circufoam**
**Process**	The PUFW is depolymerized through two successive steps: (i) reaction with a (di)carboxylic acid and a high-molecular-weight polyetherol and (ii) the overall mixture is reacted again with a short-chain di or triol. Acidolysis-glycolysis approach.^145^
**Partnership**	RetourMatras, Ikano Industry, H&S Anlagentechnik, Renewi and Ingka Group.^146,147^
**TRL**	Industrial implementation and semi-industrial scale applications (8), with a 200,000 mattresses processing capacity per year.^146,147^
**Plant location**	Lelystad, Netherlands.^146,147^
**Patents**	WO 2018/091568 A1 (H&S Anlagentechnik).^145^
	
**Consortium (3)**	**BASF-driven technologies**
**Process**	Wet-Chemical depolymerization based on a first sheared treatment of the polyurethane along with a polyhydroxy oligomer (Hydroxyl index value from 20 to 1000 mg KOH/g) to obtain a polyol dispersion at high temperatures.^148^
**Partnership**	Rigid PUF: BASF, RAMPF, KraussMaffei, REMONDIS.^149^ Flexible PUF: NEVEON.^150^
**TRL**	Pilot-scale pilot and precommercial deployment (6–7)
**Plant location**	-
**Patents**	WO 2025/030930 A1,^148^ EP 447415 A1 (BASF).^151^
	
**Consortium (4)**	**Recpur (Reciclex)**
**Process**	Acidolysis and/or alcoholysis depending on the type of foam. Characteristics of the overall process unknown.^105,152^
**Partnership**	Repsol and RAMPF Group.^153^
**TRL**	Industrial implementation and semi-industrial scale applications (8), with a 200,000 mattresses processing capacity per year.^152,153^
**Plant location**	Puertollano, Spain.^152,153^
**Patents**	EP 3590999 B1.^154^

In this context, some academic groups have centered their efforts on the study of this novel strategy. Since 2020, Gama et al. have conducted extensive studies on the acidolysis approach.^121^ This research group has investigated the effect of this depolymerization technology with different PUFW (e.g., polyester- or polyether-based polyol and viscoelastic) and DAs (e.g., succinic and phthalic acids), evaluating its operational flexibility and the suitability of the recycled polyol to produce coatings and adhesives with more robust mechanical properties.^122,123^ Notably, they confirmed that both the temperature and the PU/DA ratio are critical parameters to consider, with a respective influence of 62.4% and 31.5% on the recycled polyol hydroxyl index value.^124^ Additionally, the acid value is also affected by these two parameters, as confirmed by linear regression models with high correlation. In a recently published review, this group outlines the status of acidolysis of PU and prospects for its future.^97^

Similarly, Christopher et al. have further explored PUF acidolysis, evaluating several kinetic parameters and delving into the reaction mechanism.^104,125,126^ Using a novel and tailored shrinking core kinetic model based on CO_2_ evolution, the authors demonstrated that the DA substrate phase was the most accurate descriptor of the apparent reaction order.^125^ Moreover, they previously showed that the number of carbon atoms between the carboxyl groups was the physical parameter that best correlates with the rate constant, with maleic acid exhibiting the highest reaction rates at lower temperatures (e.g., 175 °C and 15 min).^103,127^ These conclusions provide notable information and reasoning about the acidolysis mechanism, thus progressing this novel depolymerization approach. Additionally, they carried out a thorough analysis of the most significant strategies, including hydrolysis, aminolysis, and glycolysis.^74^ Overall, both groups have contributed to the proper dissemination of this novel strategy, favoring its understanding in academia.

On the other side, Rodriguez et al. have provided the scientific community with several contributions about the glycolysis of PUFW, the understanding of the split-phase glycolysis mechanism, and scale-up processes.^128^ Their research passed through the study of the influence of different glycols and catalysts^129,130^ and continued with the valorization of crude glycerol (a biodiesel residue) through its use as an alternative transcarbamoylation agent.^131,132^ This pioneering introduction improved the sustainability and profitability of the overall process due to the reduced reliance on virgin fossil-based glycols and the higher polyol yields obtained in the upper phase, as a consequence of glycerol’s larger dielectric constant.

Moreover, most recent studies demonstrate the complete recovery and valorization of upper and bottom phases after split-phase glycolysis, as well as of the solid fillers generally present in the PUF structure (e.g., styrene–acrylonitrile and calcium carbonate).^133,134^ Split-phase glycolysis was carried out with diethylene glycol (DEG) and DABCO as nucleophile and catalyst, respectively, with a 1:1 glycol-to-PUF ratio and an optimized 0.1 wt % of catalyst. This depolymerization reaction was conducted at 200 °C for 3 h, resulting in 99% recycled polyol purity determined by Gel Permeation Chromatography (GPC), capable of substituting 100% of virgin polyol during the synthesis of new PUF. Bottom-phase byproducts (e.g., carbamate oligomers) were then subjected to hydrolysis at the same operating conditions to obtain TDA and DEG. Then, both products were separated, with TDA being used as a reagent for the synthesis of polyureas and polyamides, and DEG as a glycolysis agent for another depolymerization cycle. However, despite the promising results, the high reaction temperature and glycol mass consumption, as well as the required separation protocols (e.g., vacuum distillation), are major drawbacks to accomplish its industrial implementation.^77,135^

It is worth noting that these academic research efforts and outcomes have boosted industrial implementation through public–private partnerships and EU-backed initiatives. Among others, two Horizon 2020 projects, PUReSmart and Circular Foam, could be used as representative examples of how EU funding has supported mattress and insulation panel recycling efforts.^136–138^ The *PUReSmart* project, codirected by Recticel and Covestro, from Belgium and Germany, respectively, culminated in the construction of a pilot plant in Leverkusen for the depolymerization of discarded mattresses, based on Evocycle CQ Mattress technology, driven primarily by Covestro.^139^ Similarly, the *Circular Foam* project aims to upcycle EoL rigid PUFs derived from insulation panels used in refrigerators and construction.^138^ This consortium encompasses private entities from different industrial areas (i.e., Covestro, Interzero, REDWAVE, Sulzer, *etc*.) along with universities (e.g., ETH Zürich) and institutes (e.g., Fraunhofer Institute for Material Flow and Logistics). For both P*UReSmart* and *Circular Foam* projects, publicly available information provides insights into technological concepts, pilot-scale demonstrations, and a chronological roadmap, rather than detailed techno-economic and long-term performance data. Accordingly, these projects portray illustrative examples that link academic research and semi-industrial deployment.

However, the pursuit of a sustainable and economically viable route for PUFW depolymerization has become particularly prominent in the industry, and the scale and visibility of purely industrial initiatives now outshine the previously mentioned public-private consortia. This landscape is currently shaped by large consortia involving major chemical companies such as Dow, BASF, or Repsol.^140^ Table 3 highlights the most relevant and recent partnerships (2020 onward) regarding the chemical recycling of PUFW. These industrial technologies converge on three common operational features: (i) a mechanical pretreatment step to pulverize the PUFW into powder, maximizing the specific surface area; (ii) the use of compatible high-molecular-weight polyols as the reaction medium; and (iii) the adoption of glycolysis or acidolysis mechanisms as the main depolymerization pathways, or even a combination of both.

Preliminary estimates suggest that the largest recycling plants using these methods could process up to 200,000 mattresses per year, a substantial figure but still significantly below the 40 million mattresses that are discarded every year in the EU.^141^ This gap underscores the scalability challenges and the need for strategic industrial planning.

The industrial implementation of such new technologies implies consideration of several aspects of the process methodology. Security concerns regarding the operating conditions and environmental regulations are two key features that must be addressed, given the transitional political framework.^155^ In this sense, recent works have highlighted the relevance of combining mass- and energy-based indicators with more profound methodologies, such as LCA,^156^ particularly in low-TRL or emerging technologies. Likewise, concepts, such as prospective LCA, provide more robust and transparent decision-making toward feasible and sustainable scale-up.^157^ Similarly, to carry out an extensive TEA is essential to estimate the profitability of the overall process and, thus, its real potential.^158^

In this sense, the theoretical and computational evaluation of PUF depolymerization mechanisms with machine learning would represent a major advance, potentially accelerating the discovery and scale-up of innovative recycling technologies. Nevertheless, several authors agree that there is a critical lack of comprehensive assessments addressing the environmental and economic implications of integrating recycled polyols into new PUF formulations.^73,74,159^ In this field, only one study regarding the LCA of rigid PUFs produced with growing fractions of recycled polyols has been reported.^160^ As LCA, this evaluation encompassed raw material extraction, production, and transport, as well as foam manufacturing and EoL management. The results concluded that recycled content levels of 50–70% delivered the most favorable environmental results, thereby demonstrating that higher recycled fractions do not necessarily translate into better sustainability performance, as outcomes strongly depend on the allocation approach adopted and the final material properties.^161^ Noteworthy, this LCA considered multiple impact categories, including global warming potential (GWP), cumulative energy demand, and fossil resource depletion, within a *cradle-to-grave* system boundary for PUF production. Importantly, not all environmental indicators were simultaneously optimized, as reductions in GWP did not necessarily correlate with lower energy demand or fossil resource use. Also, the resulting sustainability rankings were highly sensitive to allocation choices, particularly in how environmental burdens were distributed between virgin and recycled material streams.

Collectively, these insights underscore the urgent need for rigorous theoretical, computational, and sustainability-oriented evaluations to fully establish the potential of recycled polyols in PUF synthesis and to guide their effective integration into industrial practice. Additionally, while full TEAs remain scarce, preliminary assessments consistently identify energy demand, solvent recovery, separation costs, and end-product performance as dominant economic drivers. Despite demonstrated technical feasibility with both high TRL and product quality, the price disparity between virgin and recycled polyols is the largest hindrance for investors to scale current recycling plants to the real magnitude of the problem, rather than remain at these absurd waste processing ratios (>1 w% of annual EU mattress waste). There is a lack of agreement among powerful lobbies on who should assume the costs derived from the collection, presorting, and dismantling of such wastes: governments, petrochemical companies, slab-stock producers, distributors, or final consumers.

## 8 Biotechnology-Based Depolymerization Processes

Biotechnology-based depolymerization approaches have emerged as promising complements or alternatives to traditional plastic recycling methods. These biological processes operate under milder temperature and pressure conditions, offering energy savings and potentially higher selectivity.^162^ This biological degradation involves either whole microorganisms (e.g., bacteria and fungi) or their isolated enzymes (see Figure 8).^35,163^

**Figure 8:**
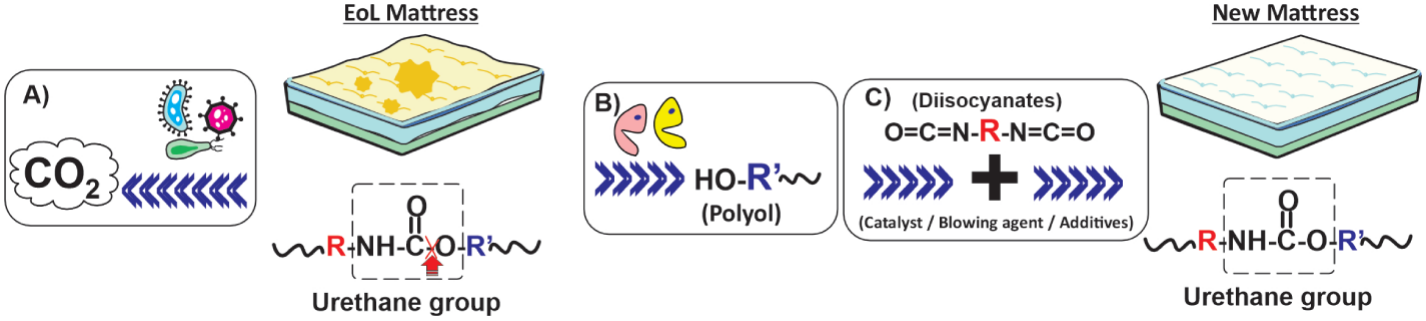
Biological depolymerization based on: A) living microorganisms, B) enzymes, and C) subsequent resynthesis of new mattresses.

From a functional standpoint, the microbial degradation of plastics under aerobic conditions results in their complete mineralization to CO_2_. Although this biological degradation effectively reduces plastic waste, the conversion of visible and localized waste (e.g., EoL plastics) into invisible and delocalized waste (e.g., CO_2_) does not facilitate the recovery of valuable intermediates or monomers, thereby limiting its contribution to a circular economy framework.^164^ In contrast, enzymatic depolymerization emerges as a feasible manner to efficiently recover the initial monomers and perform a truly circular economy.^44^ This biological depolymerization has already been successfully scaled up in less complex polymers such as PET, by using computer-aided enzyme engineering.^165^ In this field, Carbios has pioneered the industrial deployment of PET biorecycling,^166,167^ with the first commercial-scale plant under construction in Longlaville, France.^168^

Despite these successes in PET depolymerization, enzymatic recycling of PUFs remains in its infancy.^162^ Several recent studies underscore the limitations of current enzymatic systems. For example, *Humicola insolens* cutinase achieved only 42% hydrolysis of PU–polyester films after 168 h at 50 °C.^169^ Schmidt et al. reported a weight loss of 4.9% for commercial PU–polyester Impranil DLN after 200 h at 70 °C using polyester hydrolases.^170^ Similarly, *Candida rugosa* lipase generated DEG at a rate of only 0.12 mg · L^–1^· min^–1^ under aqueous conditions.^171^ Other efforts using hydrophobic ionic liquids (e.g., [Bmim]­[NTf_2_]) and enzyme mixtures yielded low conversions (e.g., 35 μmol product in 48 h at 60 °C).^172^ These approaches’ long reaction times to reach reasonable conversions represent the most prohibitive barrier to commercial enzymatic PU recycling, despite favorable operating temperatures.

Given these limitations, researchers have turned to hybrid chemoenzymatic approaches. In this context, Bornscheuer et al. made impressive advancements by discovering novel urethanases through functional metagenomic screening of soil exposed to PU waste.^173^ Their proposed two-step method begins with glycolysis of polyether-PU using DEG at >200 °C, producing a polyol-rich upper phase and a bottom phase with dicarbamates and a DEG excess. In a subsequent enzymatic step, these dicarbamates are hydrolyzed by metagenome-derived urethanases, achieving full conversion within large reaction times at moderate-to-low temperatures (e.g., 48 h and 70 °C). This enzymatic protocol was previously patented by Covestro Intellectual Property GmbH & Co. KG.^174^ However, such protocols still depend on low substrate concentrations (e.g., ∼1% w/v) and extended reaction times, likely due to enzyme instability and substrate inhibition.^175^

Subsequent work focused on testing different engineered mutants and substrates (e.g., glycolyzed products from rigid PUF), which permitted yields of up to 90% of 4,4’-methylenedianiline in 1 h for best-performing variants.^176^ However, minimal substrate loading (e.g., ∼1% w/v) and particle size (nanoscale) remain the main limitations that keep this methodology at an early technical stage. Lastly, the high-resolution crystal structures of one of these enzymes (SP3, described in previous work) at different reaction stages have enabled the identification of key amino acid substitutions (e.g., R209A) that enhance hydrolase activity.^177^

Similarly, the recent work by Chen et al. highlights that hybrid processes combining chemical recycling with biocatalysis are, at present, the only process-compatible pathway to integrate enzymes into PUFW management at scale. In this sense, the authors managed to valorize the bottom phase of a kilogram-scale split-phase glycolysis process with a DEG-tolerant urethanase without solvent exchange or enzyme immobilization. Results showed >98% conversion at 50 °C within 8–12 h, with a recovery of up to 94.7% and 98.5% w/w of TDA and DEG, respectively.^178^

Overall, enzymatic depolymerization of PUF is far from industrial readiness. The high polymer complexity, hydrophobicity, and recalcitrance (e.g., *N-*aryl *O-*alkyl carbamate bonds) hamper interaction between the active site of the enzyme and the substrate.^162,179,180^ Moreover, the release of toxic monomers (e.g., aromatic (di)­amines, precursors of isocyanates) entails another relevant drawback.^180^ This outlines the necessity of combining machine learning, protein engineering, and site-specific mutagenesis to rationally improve enzyme thermostability, substrate specificity, and catalytic efficiency,^35,177^ eventually unlocking new biocatalytic pathways for PUF depolymerization. While hybrid chemoenzymatic routes are promising, the energy demands of chemical pretreatment and the long reaction times of the subsequent biocatalytic stage reduce their overall environmental advantage. This underscores the current prevalence of chemical depolymerization pathways for PUF recycling.

Nevertheless, the rapid pace of innovation in protein engineering, machine learning, and industrial biotechnology suggests that biocatalytic depolymerization could eventually play a meaningful role in closing the loop for thermoset plastics, unlocking a new frontier in the circular polymer economy. However, continued investment and research in enzymatic pathway optimization and hybrid systems design are key to confirm whether this biotechnology-based recycling represents a key solution or remains a transitory technological trend.

## 9 Future Trends: Biobased Products and Dynamic Systems

While the development of efficient EoL strategies represents a crucial step toward improving the sustainability of PUF-based products, it only addresses one part of the broader challenge. To achieve a truly sustainable PU value chain, several parameters encompassing the sourcing, design, and synthesis of both the raw materials and final products must be considered. This field includes both biobased synthesis of products and carbon capture and utilization (CCU) strategies.^181,182^ However, although each technology presents unique opportunities to reduce dependence on fossil-derived resources and minimize environmental impact, their technological maturity, scalability, and environmental trade-offs differ substantially.

The use of biobased polyols derived from renewable feedstocks (i.e., vegetable oil-derived, lignocellulosic biomass) is technically feasible and could reduce reliance on fossil resources.^183^ Examples include the use of long-chain liquid polyols from soybean or castor oils,^184^ glycerol derivatives from biodiesel production,^185^ or lignin-based aromatic polyols.^186^ However, such inputs raise concerns regarding land usage competition with the food industry^187^ and the planetary boundaries framework (i.e., biosphere integrity, N cycle footprint, *etc*.).^48^

Likewise, CCU technologies have arisen as a carbon-neutral alternative to traditional feedstocks. However, these systems heavily rely on hydrogen produced *via* water electrolysis, a process that demands a sizable energy input. Consequently, the environmental viability of CCU-based routes hinges on the availability of low-carbon electricity sources, such as wind or solar energy.^188^ In this context, hybrid systems integrating CCU with biobased synthesis have been proposed to mitigate this energy dependence.^189^

A particularly promising innovation enclosed within these “hybrid systems” is the development of nonisocyanate polyurethanes (NIPUs).^190^ These materials eliminate the use of toxic isocyanates by utilizing cyclic carbonates and amines to form urethane linkages through ring-opening polymerization. This approach not only minimizes health and safety concerns but also enables the use of renewable carbon sources, such as CO_2_-derived carbonates^191^ and/or biobased amines.^192^ However, current limitations may include the limited mechanical properties of the final product and scalability, as well as the difficulties owing to the sustainable synthesis of precursors (i.e., CO_2_ cycloaddition, epoxide synthesis, biobased amine formation).^193–195^ In this sense, ongoing innovations in monomer synthesis and catalysis are narrowing this gap, slightly positioning NIPUs as a viable route toward greener PU chemistry.^196^

Looking ahead, Dynamic Covalent Polymer Networks (DCPNs) are emerging as a promising tool in the recent push toward “rethink-the-industry.” ^197,198^ This scientific and industrial movement is challenging the conventional, linear paradigm of polymer design and manufacturing, aiming instead to integrate circularity, sustainability, and functionality at the molecular level, and placing dynamic bonds at the core of the material architecture.^106^ In the PUF context, recent advances have demonstrated the feasibility of incorporating such dynamic covalent motifs into the molecular cross-linked network. Notably, polyhydroxyurethane networks, formed by the ring-opening of cyclic carbonate with primary amines, feature a 1:1 ratio of hydroxyl to carbamate groups. This proximity of hydroxyls to carbamates enhances both stress relaxation and bond exchange, enabling reprocessability.^199^ Besides, the synthesis of Dynamic Covalent Bonds through isocyanate-involved chemistry has emerged as a feasible contribution to a circular economy in the PU industry, mainly due to the reversibility of these networks governed by electron-withdrawing, delocalization, and steric effects.^200^ In another work, Zhou et al. prepared a thermoset PU double-locked covalent adaptable network (DLCAN) combining a castor oil-derived isocyanate with imine and disulfide linkages, demonstrating closed-loop behavior by applying programmed stimuli.^201^

These dynamic frameworks offer promising proof-of-concept results; however, they remain at a low TRL due to issues related to (i) limited mechanical robustness arising from reversible bonding, (ii) elevated activation temperatures incompatible with service conditions, and (iii) reliance on nonstandard monomers or catalysts, among others.^198,202^

## 10 Conclusions

The transition from a linear “take-make-dispose” model toward a circular economy for PUFs remains an urgent and multifaceted challenge. This review highlights key drivers, technological advances, and systemic limitations that shape the future of PUF sustainability.

First, among recycling technologies, chemical depolymerization—particularly glycolysis and acidolysis—emerges as the most mature and industrially viable solution for addressing the inherent limitations of mechanical recycling in thermoset polymers. These approaches effectively recover high-quality monomers, reduce reliance on virgin fossil-based inputs, and enable closed-loop production systems. Industrial consortia, such as the RENUVA program, demonstrate the scalability and commercial readiness of these strategies. Similarly, public-private partnerships, supported by EU initiatives (e.g., Circular Foam), aim to decouple economic growth from resource consumption by transitioning toward circular economy solutions.

Second, despite the polarized discourse within academia between glycolysis and acidolysis, this article demonstrates that there are no conclusive data supporting the prevalence of one depolymerization technology over the other. This is particularly evident when examining industrial-scale PUF recycling plants, which frequently integrate both mechanisms in series, or their use differs according to factors such as the nature of the substrate or the final application of the recycled polyol.

Third, although biotechnology-based processes, such as enzymatic depolymerization of PUFs, offer long-term potential, they are not feasible yet at the industrial scale. Enzyme instability, long reaction times, and the need for harsh chemical pretreatments limit their near-term applicability.

Fourth, achieving full circularity requires extending sustainability principles upstream toward the synthesis of the monomers themselves. In this context, biobased polyols, CCU, and NIPUs approaches, while promising, are constrained by scalability, energy intensity, and feedstock competition. Similarly, although DCPNs enable easy disassembly, reprocessing, and repolymerization, their low TRL regarding scalability, cost, and long-term performance suggests that their industrial deployment remains a mid- to long-term perspective rather than an immediate solution. Overall, these emerging technologies must be envisioned as complementary and not alternatives to chemical recycling for the foreseeable future.

Fifth, PUFW management remains a systemic issue involving complex materials, additive-rich formulations, and challenging EoL logistics. LCA studies, although still rare in this domain, will be essential to verify environmental benefits and to guide process optimization. Efforts to combine technical innovation and material performance analysis (e.g., TEA) with environmental metrics, such as prospective LCA, will be key to achieving sustainable scale-up.

Ultimately, the success of any recycling strategy will depend not only on technological advancement but also on regulatory alignment, cross-sector collaboration, and societal engagement. The absence of binding legislation and the overreliance on voluntary initiatives have, thus far, limited the circularity outcomes. Real progress requires coherent policy frameworks, greater accountability across the value chain, and increased consumer awareness to shift the demand toward sustainable products.

As emphasized throughout this review, the core challenge no longer lies solely in chemistry, innovation, or social awareness, but rather in confronting the economic and political inertia that dictates the pace at which any meaningful environmental change can truly happen.

## Abbreviations

LCA: Life-Cycle Assessment
TEA: Techno-Economic Analysis
EoL: End-of-Life
PUF: Polyurethane Foam
TRL: Technology Readiness Level
CAGR: Compound Annual Growth Rate
PVC: Polyvinyl Chloride
EPS: Expanded Polystyrene
XPS: Extruded Polystyrene
VPR: Virgin Plastic Reduction
PCRc: Post-Consumer Recycled content
RP: Reusable Packaging
R/C: Recyclable/Compostable
PPR: Problematic Plastics Removal
PP: Polypropylene
HDPE: High-Density Polyethylene
LDPE: Low-Density Polyethylene
LLDPE: Linear Low-Density Polyethylene
PET: Polyethylene Terephthalate
PLA: Poly­(lactic acid)
FDA: Food and Drug Administration
PU: Polyurethane
PUFW: Polyurethane Foam Waste
CSIL: Center for Industrial Studies
DABCO: 1,4-Diazabicyclo­[2.2.2]­octane
EU: European Union
USA: United States of America
DAs: Dicarboxylic Acids
DEG: Diethylene Glycol
TDA: Toluene Diamine
GPC: Gel Permeation Chromatography
CCU: Carbon Capture and Utilization
NIPUs: Nonisocyanate Polyurethanes
DCPNs: Dynamic Covalent Polymer Networks
DLCAN: Double-Locked Covalent Adaptable Network
GWP: Global Warming Potential
